# Salinity drives meiofaunal community structure dynamics across the Baltic ecosystem

**DOI:** 10.1111/mec.15179

**Published:** 2019-09-05

**Authors:** Elias Broman, Caroline Raymond, Christian Sommer, Jonas S. Gunnarsson, Simon Creer, Francisco J. A. Nascimento

**Affiliations:** ^1^ Department of Ecology, Environment and Plant Sciences Stockholm University Stockholm Sweden; ^2^ Baltic Sea Centre Stockholm University Stockholm Sweden; ^3^ School of Natural Sciences, Technology and Environmental Studies Södertörn University Huddinge Sweden; ^4^ Molecular Ecology and Fisheries Genetics Laboratory School of Natural Sciences Bangor University Bangor UK

**Keywords:** community ecology, DNA barcoding, population dynamics, population ecology

## Abstract

Coastal benthic biodiversity is under increased pressure from climate change, eutrophication, hypoxia, and changes in salinity due to increase in river runoff. The Baltic Sea is a large brackish system characterized by steep environmental gradients that experiences all of the mentioned stressors. As such it provides an ideal model system for studying the impact of on‐going and future climate change on biodiversity and function of benthic ecosystems. Meiofauna (animals < 1 mm) are abundant in sediment and are still largely unexplored even though they are known to regulate organic matter degradation and nutrient cycling. In this study, benthic meiofaunal community structure was analysed along a salinity gradient in the Baltic Sea proper using high‐throughput sequencing. Our results demonstrate that areas with higher salinity have a higher biodiversity, and salinity is probably the main driver influencing meiofauna diversity and community composition. Furthermore, in the more diverse and saline environments a larger amount of nematode genera classified as predators prevailed, and meiofauna‐macrofauna associations were more prominent. These findings show that in the Baltic Sea, a decrease in salinity resulting from accelerated climate change will probably lead to decreased benthic biodiversity, and cause profound changes in benthic communities, with potential consequences for ecosystem stability, functions and services.

## 1 INTRODUCTION

Biodiversity underpins essential ecosystem services for human benefits such as food availability, provision of clean water, recreational areas and activities affiliated with human health, and play key roles in ecosystem processes such as nutrient cycling and secondary production (Pan, Marcoval, Bazzini, Vallina, & Marco, 2013). Climate change, eutrophication with associated algal blooms, hypoxic bottom zones, and changes in salinity are contemporary major threats for coastal biodiversity (Pan et al., 2013). Such impacts need to be understood in order to predict how marine ecosystems will respond to future changes.

The Baltic Sea is a brackish water system that contains strong abiotic environmental gradients in salinity, depth and temperature that structure its biodiversity and benthic community structure (Ojaveer et al., 2010). The Baltic Sea is also affected by multiple anthropogenic pressures like eutrophication (Conley, 2012) and climate change (Vuorinen et al., 2015). In its deeper basins, below the halocline, hypoxic and anoxic benthic zones are widespread (Conley, 2012). Low‐saline areas (<6 ppt) have expanded in the Baltic Sea since the 1970s and are predicted to further increase with climate change due to increased freshwater runoff and increased water column stratification (Vuorinen et al., 2015). The Baltic Sea therefore presents an ideal ecosystem to study the impact of future climate change scenarios on biodiversity (Ojaveer et al., 2010) and concomitant effects on benthic structure and consequent benthic‐pelagic coupling (Griffiths et al., 2017). Most knowledge on how benthic organisms in the Baltic Sea react to these pressures are based on benthic macrofauna, while meiofauna (animals < 1 mm) have been studied much less. Meiofauna is a much more abundant and diverse metazoan group in sediments than macrofauna and plays an important role in a number of ecosystems process (Bonaglia, Nascimento, Bartoli, Klawonn, & Brüchert, 2014; Nascimento, Näslund, & Elmgren, 2012; Näslund, Nascimento, & Gunnarsson, 2010). However, there are still large knowledge gaps regarding how meiofaunal diversity and structure is affected by environmental changes (Bik et al., 2012). Recent DNA and RNA techniques now offer new possibilities to better address such questions on larger geographical scales than previously possible with traditional techniques.

Meiofauna have a short life span and are known to stimulate bacterial growth (reviewed in Coull & Chandler, 2001). Meiofaunal diversity and community composition are structured by several interacting factors; both abiotic and biotic (Giere, 2009). Oxygen is important for meiofaunal survival and metabolism (Braeckman, Vanaverbeke, Vincx, van Oevelen, & Soetaert, 2013), with some exceptions for facultative anaerobes with anaerobic mitochondria (Tielens, Rotte, van Hellemond, & Martin, 2002). Additionally, meiofaunal species richness and abundance have been found to increase with increasing salinity (Coull, 1988). In benthic environments these organisms rework sediment particles through e.g., bioturbation (Cullen, 1973), and have been found to affect porosity and increase the transport of solutes in the sediment (Aller & Aller, 1992). Meiofauna utilize many sources of organic substrates in the lower trophic food web, e.g., bacteria, and detritus such as settling algal matter from the pelagic water (reviewed in Schratzberger & Ingels, 2018). Furthermore, they have also been found to stimulate degradation of sediment organic matter (OM) and bacterial denitrification (Bonaglia et al., 2014), and may therefore be key players in sediment habitats influencing carbon and nitrogen cycles.

One of the most diverse animal groups on Earth are the roundworms, i.e., nematodes (Zhang, 2013), and they are also one the most abundant meiofauna in sediments (Coull, 1999). Nematodes have been found to enhance the oxygen production in diatom biofilms (Mathieu, Leflaive, Ten‐Hage, De Wit, & Buffan‐Dubau, 2007), and to enhance the mineralization of OM (Nascimento et al., 2012). Because of their different feeding behaviours in sediments, nematodes have been widely used in functional analyses (e.g., Semprucci, Cesaroni, Guidi, & Balsamo, 2018; Vanaverbeke, Merckx, Degraer, & Vincx, 2011). An increased knowledge of nematode community composition in the Baltic Sea could therefore further elucidate the role of trophic interactions in sediments under anthropogenic stress and climate change scenarios.

Benthic macrofauna have been observed to control meiofauna populations (or limit in some cases) through for example predation (Olafsson, 2003) and competition of limited resources (Ingels, Dashfield, Somerfield, Widdicombe, & Austen, 2014; Nascimento, Karlson, Näslund, & Elmgren, 2011; Olafsson, 2003). There has been extensive work, mainly laboratory or in situ experimental approaches, conducted on meiofauna‐macrofauna interactions using morphological approaches (Olafsson, 2003). Such studies have yielded a variety of mixed results, but also a general consensus that macrofauna bioturbation structures the meiofauna community (Olafsson, 2003). These ecological interactions have been shown to have an importance on biogeochemical cycles; however, studies that focus on meiofauna‐macrofauna interactions in situ and over regional and ecologically relevant scales are scarce. Macrofauna diversity is generally higher in more saline regions (Gogina et al., 2016), and meiofauna‐macrofauna interactions might therefore be more prominent in saline regions with higher diversity and species richness. Gaining such insights will help to elucidate potential trophic interactions in the sediment and how these may be affected by contemporary ecological and environmental pressures.

Studies using metabarcoding, i.e., high‐throughput sequencing of taxonomically‐informative marker genes, to investigate meiofaunal biodiversity is a growing field (Bik et al., 2012; Carugati, Corinaldesi, Dell'Anno, & Danovaro, 2015; Fonseca et al., 2010; Lallias et al., 2014; Peham, Steiner, Schlick‐Steiner, & Arthofer, 2017), and opportunities to facilitate such insights and the investigation of 18S rRNA gene meiofauna community in the Baltic Sea are now emerging (Nascimento, Lallias, Bik, & Creer, 2018). Compared to traditional morphological taxonomic techniques, modern sequencing tools facilitate the study of regional patterns of meiofauna diversity in less time while requiring no specific expertise in morphological taxonomy (Carugati et al., 2015). However, caveats do exist, such as not being able to determine absolute abundance and limitations of reference databases to assign taxonomy (Carugati et al., 2015). The benthic meiofauna community of the Baltic Sea is still largely unexplored although many benthic habitats in the Baltic Sea are under stress from anthropogenic pressure.

In this study we aimed to assess Baltic Sea meiofaunal diversity and community structure at the ecosystem level. An additional goal was to improve our understanding of possible future trajectories of benthic coastal diversity by using the Baltic Sea as a model system. We specifically tested the following hypotheses: (a) salinity is an important driver of meiofauna community structure in the Baltic Sea, and (b) biotic interactions with macrofauna play a more important role in structuring meiofauna communities in more saline areas coincident with higher macrofaunal species richness. To test these hypotheses we sampled sediment along a salinity gradient in the central Baltic Sea (Baltic Proper). In order to identify changes in community composition and diversity of benthic taxa, a combination of traditional taxonomic assessment for macrofauna and metabarcoding DNA analyses for meiofauna were used. Meiofauna community composition was then analysed together with macrofauna community composition and sediment abiotic parameters (sediment water and OM content, bottom water temperature, salinity, and dissolved oxygen). Finally, because of the large relative abundance and diversity of nematodes, data for the phylum Nematoda were analysed separately to investigate their functional ecology (maturity index and feeding type) along the salinity gradient.

## 2 MATERIALS AND METHODS

### 2.1 Field sampling, collection of macrofauna, and abiotic variables measurements

**Figure 1:**
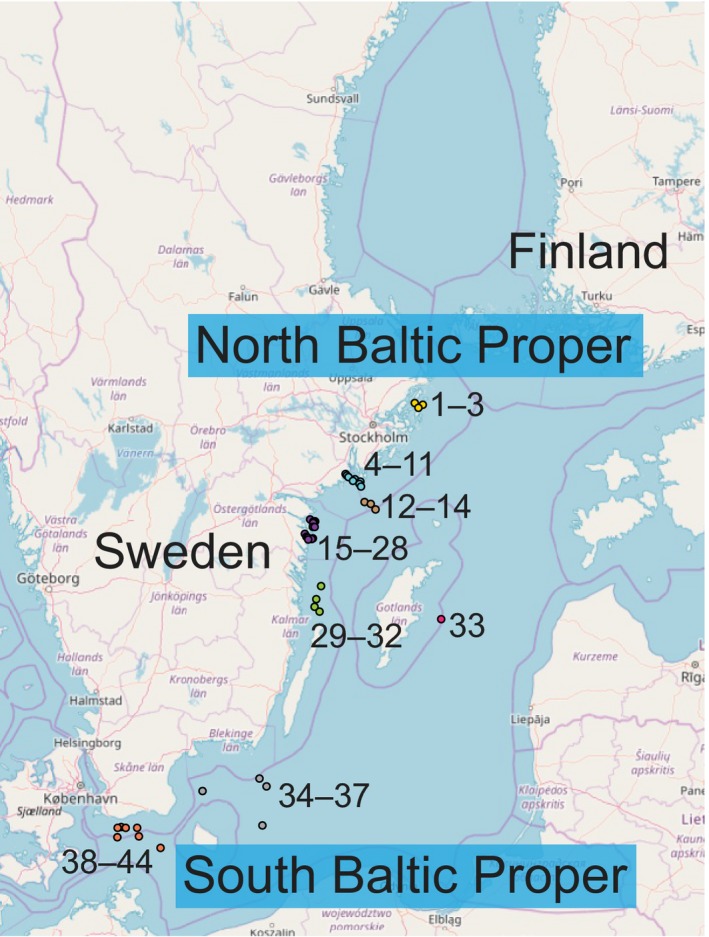
The figure shows a map of the Baltic Sea and each sampling station and geographical regions (different coloured circles). Full names and details of the sampling stations are presented in Table 1. The Baltic Proper was divided into two areas for this study: the north Baltic Proper (NBP; stations 1–33) and the south Baltic Proper (SBP; stations 34–44). The colours of the circles denote the different regions in the study, with: yellow as Stockholm; light blue Sörmland; brown Sörmland offshore; purple Östergötland; green Västervik; red Gotland (one station only); grey Bornholm; and orange as Arkona. The map layer is © OpenStreetMap contributors, CC BY‐SA [Colour figure can be viewed at http://wileyonlinelibrary.com]

Soft bottom sediment of similar clay‐muddy habitats and water samples were collected in May‐June 2015, at 44 stations in the Baltic Sea from the Stockholm region to the southern Arkona basin proper, during the yearly Swedish national and regional benthic monitoring program (Figure 1). Benthic macrofauna communities were sampled with a van Veen sediment grab (0.1 m^2^) from each station (typically one replicate per station, except for nine stations that had three replicates due to a yearly monitoring programme: 4, 5, 8, 11, 13, 14, 33, 37, and 44). All macrofauna abundance and biomass data were normalized for m^2^ sediment. Benthic meiofauna and sediment variables were measured by collecting sediment cores from the 44 stations using a Kajak gravity corer (surface area: 50 cm^2^, one core per station). To investigate large spatial scale variation, we sampled more stations within each region, rather than performing repeat sampling within stations. The latter strategy has been demonstrated to be effective at capturing both small and large spatial scale diversity of European meiofaunal communities (Fonseca et al., 2014; Lallias et al., 2014). Consequently, sediment collected from stations within the same region were treated as ecological replicates for further analyses. For the meiofauna and sediment organic matter the top 0–2 cm layer of each sediment core was sliced and homogenized into a clean and rinsed 215 ml polypropylene container (207.0215PP; Noax Laboratory). Sampling and slicing equipment was rinsed with deionized water between each sample. The sliced portion was then divided into: (a) 15 ml transferred to a 90 ml polypropylene container (207.0090PP; Noax Laboratory) for measurement of water and OM content, and (b) the remaining portion kept for meiofauna extraction. Samples were frozen at –20°C while on the boat, put on ice during transportation to the laboratory (~2 hr), and finally stored at –20°C until DNA extraction. Sediment collected for macrofauna was sieved through a 1 mm mesh and the animals retained in the sieve were transferred to 100–1,500 ml polypropylene containers (Noax Laboratory) and conserved in 4% buffered formaldehyde for three months (EN 16655:2014, 1992). Macrofauna abundance and wet weight biomass were counted visually and weighed according to the European standard (EN 16665:2014: 1992). Sediment water content (%) and OM content (%) were analysed according to Dybern, Ackefors, and Elmgren (1976). In more detail, determination of water content was conducted by drying sediment at 80°C to a constant weight (at least for 12 hr, typically overnight). The OM content was measured by reweighing the dry sediment after loss on ignition (500°C for 2 hr). Bottom water was sampled at each station, approximately 20 cm above the sediment surface, with a modified Niskin bottle. On deck temperature and salinity were measured in the collected bottom water using a digital multimeter (WTW Cond 340i), and dissolved oxygen (O_2_) was measured in duplicate samples using the Winkler titration method (EN 25813:^1992^).

### 2.2 Collection of meiofauna, DNA extraction, and sequencing

The sediment collected for meiofauna analysis was thawed at the laboratory and meiofauna were extracted from the sediment using the procedure described by Nascimento, Karlson, and Elmgren (2008). Sediment samples were sieved through a sterilized 40 µm sieve (autoclaved, rinsed with 90% ethanol and MilliQ water between samples). Meiofauna retained on the 40 µm sieve were isolated by density extraction using a Levasil silica gel colloidal dispersion solution (H.C. Starck) with a density of 1.3 kg/m^3^. The isolation was performed by shaking an Erlenmeyer flask with sediment and Levasil and let it stand for 5 min, while the sediment particles settle and the meiofauna floats up. The top part of the solution containing the meiofauna was decanted and washed with seawater (of approximately equal salinity to the respective sampling site). This isolation procedure was repeated twice (a second isolation with 5 min of settling time, followed by a third and final isolation with 30 min of settling time). The pooled content of these three isolations was then placed in the 40 µm sieve and washed thoroughly with seawater to remove any remaining Levasil. The 40 µm sieve content was transferred into a 50 ml falcon tube with a maximum final volume of 10 ml meiofauna isolate (representing the total meiofauna individuals from approximately 100 g of wet sediment). The meiofauna isolate was then frozen at –20°C until DNA extraction.

DNA from the meiofauna isolate was extracted with the PowerMax Soil DNA Isolation Kit (Cat#12988; MOBIO). After DNA extraction, samples were frozen at –20°C in 3 ml of elution buffer C6 solution (10 mM Tris). Following this procedure, 100 μl of each DNA extract was purified with PowerClean Pro DNA Clean‐Up Kit (Cat# 12997‐50; MOBIO) and stored in 100 µl of elution buffer C5 (10 mM tris) solution at –20°C. All DNA extracts were standardized to a concentration of 10 ng/µl before amplification. The conservative metabarcoding primers TAReuk454FWD1 (5′‐CCAGCA(G/C)C(C/T)GCGGTAATTCC‐3′) and TAReukREV3 (5′‐ACTTTCGTTCTTGAT(C/T)(A/G)A‐3′) (Stoeck et al., 2010) were used with Q5 HS High‐Fidelity Master Mix (2×) (New England Biolabs) to amplify by PCR the 18S rRNA gene region, targeting fragments between 365 and 410 bp excluding adaptors and barcodes. Each sample was amplified in triplicates, which were then pooled, dual‐barcoded with Nextera XT index primers following Bista et al. (2017) and visualized by gel electrophoresis. The barcoded amplicons were then purified with the Agencourt AMPure XP PCR Purification kit (Beckman Coulter), quantified with Qubit (Invitrogen) and pooled into a library with equimolar quantities. See full details of the PCR protocol and programs in Appendix S1. The library was sequenced with a 2 × 300 bp paired‐end setup on the Illumina MiSeq platform at the National Genomics Institute (NGI‐Stockholm).

### 2.3 Bioinformatics

A total of 18.4 million sequences, averaging 419,238 paired‐end reads per sample (44 samples), were processed following the DADA2 pipeline according to Callahan et al. (2016). DADA2 uses a parameterized model of substitution errors to differentiate between sequencing errors and biological variation. It avoids constructing operational taxonomic units (OTUs), inferring instead sequence variants. Following the DADA2 pipeline the raw sequences were trimmed to remove low quality bases (the first 10 nucleotides and from position 190 and 240), filtered (maximum of two expected errors per read), followed by merging the paired‐ends. After this procedure chimeras were then removed from the data set. Following quality filtering and chimera removal a total of 3,309 amplicon sequence variants (ASVs) and 9.2 million sequences were retained, averaging 209,545 reads per sample (minimum = 45,729 reads and maximum = 391,690 reads).

Because the taxonomic classification results from silva 132 could not satisfactorily annotate sequence variants to genus level (e.g., no Nematode sequence could be classified further than Order, as well as some sequences were incorrectly classified as Arthropoda as seen previously; Holovachov, Haenel, Bourlat, & Jondelius, 2017), the DADA2 sequence variants were additionally aligned and annotated against the NCBI NT database using blast 2.7.1+ (Altschul, Gish, Miller, Myers, & Lipman, 1990) with a 0.001 e‐value threshold and ‐max_target_seqs 1 to only report the top hit. The NCBI NT accession numbers for each sequence were imported into megan 6 (with default LCA parameters; Huson & Mitra, 2012) in conjunction with the “accession to taxonomy June 2018” megan database (nucl_acc2tax‐Jun2018.abin). This made it possible to retrieve taxonomy names based on NCBI accession numbers, and estimate more specific taxonomy with the use of the Lowest Common Ancestor (LCA) algorithm (Huson, Auch, Qi, & Schuster, 2007). The function “read names to taxonomy path” was used to extract all assigned DADA2 sequences with their affiliated taxonomy path. These results were then combined with the DADA2 sequence variants counts, and the results based on the NCBI NT database were used for taxonomy analyses. Sequences affiliated with Metazoa in the taxonomic description were extracted from the data set and analysed further as relative abundances (i.e., [*x*/sum] × 100) in the software explicet 2.10.5 (Robertson et al., 2013). In addition, Nematoda sequences were extracted into a subdata set (on average 27,385 sequence counts per sample) and phylogenetically placed on a reference tree as suggested by Holovachov et al. (2017). In more detail, reference sequences from Holovachov et al. (2017) were downloaded from NCBI GenBank, and aligned in mega 7 (Kumar, Stecher, & Tamura, 2016) using muscle (Edgar, 2004) (with settings: gap open: –400, gap extend: 0, max iterations: eight, cluster method: UPGMA, min diagonal length: 24). The alignment was used to construct a phylogenetic maximum likelihood tree with 100 bootstraps (settings: Tamura‐Nei model, nucleotide substitution type, rates among sites: uniform rates, gaps/missing data: complete deletion, ML Heuristic model: Nearest‐Neighbour‐Interchange). The Nematoda sequences were phylogenetically aligned using papara 2.5 (Berger & Stamatakis, 2011) with the constructed reference alignments and maximum likelihood tree. The output alignments were used with raxml 8.2.12 (Stamatakis, 2014) to predict the taxonomy of the aligned Nematoda sequences (with the following commands: ‐f v ‐m GTRCAT), that adds the input sequences on a reference tree using thorough read insertion with a nucleotide General Time Reversible model. The final tree was visualized in the software figtree version 1.4.3.

### 2.4 Statistics

To detect differences in community composition between sites nonmetric multidimensional scaling (NMDS) ordination was performed by loading Metazoa sequence variants data into the r package phyloseq 1.24.2 (McMurdie & Holmes, 2013) using r 3.5.1 (R Core Team, 2013). In more detail, NMDS plots of Bray‐Curtis dissimilarity, based on the sequence variants relative abundances and presence/absence (Sørensen), were constructed using the “ordination” and “plot.ordination” functions in phyloseq. To test for statistical differences in community composition, this was followed by statistical testing with pairwise PERMANOVA tests (9,999 permutations) using the adonis function in the vegan package (Oksanen et al., 2018). In addition, the “betadisper” function in the vegan package was used to find differences in multivariate homogeneity of beta diversity variance between regions (Anderson, Ellingsen, & McArdle, 2006). This was followed by PERMANOVA tests of the homogeneity variance between regions, and plotted using ggplot2 package as the average distance to the centroid. Alpha diversity indexes (ACE, Chao1, and Shannon's *H*) were based on all Metazoa sequence variations counts and were calculated in the software explicet. Before alpha diversity analysis, counts were subsampled to 2,200 counts for each station (lowest sample size; Station 14), except for one station (Station 33 Gotland) that was excluded due to having fewer counts than the amount of metazoan sequence variants in the data set (station 33:291 counts). Afterwards the data set was bootstrapped 100 times, alpha diversity was calculated, and the mean of each alpha diversity index reported. In addition, ACE alpha diversity was also calculated by using nonsubsampled counts using the fossil 0.3.7 package (Vavrek, 2011) in r.

Based on classified nematode genera that could be annotated according to functional traits, (a) the maturity index described by Bongers (1990) was calculated to identify habitat colonizers or persisters (based on a 1–5 scale per genera; values closer to 1 indicate colonizers), and (b) feeding type was determined according to Wieser (1953) for each genera based on available literature outlining their buccal cavity morphology. Statistics on alpha diversity, taxonomic groups, and nematode feeding types were conducted in the software IBM spss Statistics 25. The normality distribution of the data was tested with Shapiro Wilk tests, and nonparametric Mann–Whitney U and Kruskal–Wallis tests were used on data not following a normal distribution.

The function “bioenv” in the r package vegan was used to test which, or combination of, abiotic variables (based on euclidean distances) had the highest rank correlation explaining the Bray‐Curtis dissimilarity distribution of sequence variants among the sampling stations (with the following parameters: method = “spearman”, index = “bray”, partial = NULL, metric = c(“euclidean”)). This was followed by Mantel tests (Mantel, 1967) of Bray‐Curtis dissimilarity distances and abiotic variables (salinity and spatial distance) in r using the ade4 package and 9,999 permutations (Dray & Dufour, 2007).

To find potential biotic interactions between meiofauna and macrofauna, co‐occurrences among meiofauna, and possible community niches based on abiotic variables we conducted correlation network analysis (Röttjers & Faust, 2018). Correlation network analysis was conducted by importing Metazoa genera sequence counts as primary data, and the measured values for abiotic variables and macrofauna abundances per sediment per m^2^ as metadata using conet 1.1.1 (Faust & Raes, 2016) and visualized in cytoscape 3.6.1 (Shannon et al., 2003). The setup in conet consisted of normalizing sequence counts as proportions per sample; setting spearman correlations with *ρ* thresholds ≤–0.7 or ≥0.7, and Fisher's *z* *p*‐value threshold <.05 with Bonferroni adjustment for multiple‐test correction. We are aware that our data set included a complicated setup, less commonly used in network software (Röttjers & Faust, 2018), with 18S rRNA gene sequencing data combined with both abiotic and macrofauna data. However, we applied a number of recommendations outlined in Röttjers and Faust (2018) to minimize potential limitations of such an approach, namely: (a) data from meiofauna were physically isolated from sediments; (b) the DADA2 methodology that incorporates denoising algorithms was employed; (c) metazoan sequence variants were grouped into 125 groups (120 genera and five unclassified groups); and (d) differences in meiofaunal community composition between north and south sample regions were based on the NMDS Bray‐Curtis. In combination with the bioenv analysis that identified salinity as a major factor of diversity and community structure, we divided the data into two clusters (north and south Baltic proper) to remove influences of heterogeneous local environmental factors. Such precautions strengthen the correlation network analysis, and emphasises ecological relevance (as reviewed in Röttjers & Faust, 2018).

## 3 RESULTS

The DADA2 analysis of the raw sequence data resulted in 3,309 18S rRNA gene sequence variants of which 770 belonged to the Metazoa kingdom distributed over 120 genera. On average 23% of the sequences per sample were unassigned with blast, and could not be classified to a phyla in the silva database, and were therefore not included in further analyses. See Table S1 for a list of all DADA2 sequence variants, the taxonomic classifications and sequence counts, and Table S2 for a full list of metazoan genera.

### 3.1 Meiofauna beta and alpha diversity

**Figure 2:**
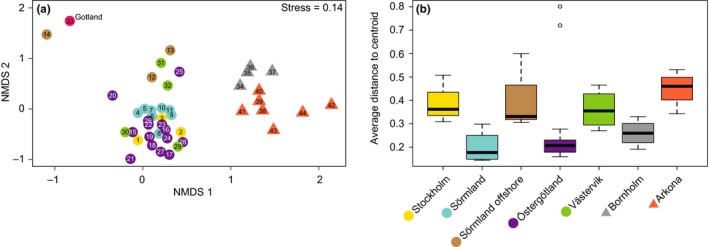
Multivariate NMDS based on the relative abundance Bray‐Curtis dissimilarities were constructed based on all sequence variants classified as meiofauna (i.e., metazoan 0.40–1,000 µm) in the 0–2 cm sediment surface layer (a), and boxplots showing the homogeneity of beta diversity variance for each region (b). The colours of the symbols in the NMDS plots denote the specific regions (as shown in Figure 1), while the numbers denote each specific station. Stations belonging to the north Baltic Proper are presented as circles while stations in the south as triangles [Colour figure can be viewed at http://wileyonlinelibrary.com]

The NMDS analysis of all meiofauna Metazoa sequence variants (based on relative abundances) showed that the majority of the sampling sites formed two significantly different clusters; one for sites located in the north Baltic Proper (from here on abbreviated as NBP, *n* = 33) and a second cluster for the south Baltic Proper (abbreviated as SBP, *n* = 11; Figure 2a; adonis, PERMANOVA tested for the two clusters, *R* ^2^ = 0.35197, *F* = 22.812, *p* < .01). Data based on presence/absence showed similar results with the two NBP and SBP clusters being significantly different (Figure S1). PERMANOVA tests also showed a difference between the sampling regions when tested with relative abundance and presence/absence for the whole model (*R* ^2^ = 0.54185, *F* = 6.0825 and *R* ^2^ = 0.46939, *F* = 4.5495, respectively; *p* < .01 for both). Looking more closely at the homogeneity of beta diversity variance between the regions in the Baltic Proper, Sörmland was significantly lower from all regions except Östergötland and Bornholm (betadisper, PERMANOVA, *p* < .01; Figure 2b, see Table S3 for a full list of *p*‐values for the geographic regions). In addition, the two regions in the SBP were significantly different from each other (i.e., Bornholm being lower compared to Arkona; betadisper, PERMANOVA, *p* < .01; Figure 2b). There was a relatively large abundance of pelagic Arthropoda in the 18S rRNA gene data set, and therefore, NMDS analysis was also performed without these sequence variants (mainly pelagic Copepod genera *Eurytemora* and *Temora*; see Table S3 for a full list of excluded genera). This analysis also showed two distinct clusters between the NBP and SBP (Figure S2a; station 33 Gotland excluded to keep statistical power, as it only contained pelagic Arthropoda; adonis, PERMANOVA, *R* ^2^ = 0.23126, *F* = 11.732, *p* < .01). After removing the pelagic Arthropoda there were more significant differences in homogeneity of beta diversity variance between regions. For example Sörmland and Östergötland were significantly different compared to all regions except Stockholm and Arkona, respectively. The deeper (64–124 m) regions Sörmland offshore and Bornholm were lower compared to all other regions. Furthermore, similar to the results from the whole data set the southern region Bornholm was significantly lower compared to the other southern region Arkona (betadisper, PERMANOVA, *p* < .05 for all tests; Figure S2b and Table S3). As such, the differences in meiofaunal homogeneity variance between regions were larger after the pelagic Arthropoda had been excluded from the data set.

**Figure 3:**
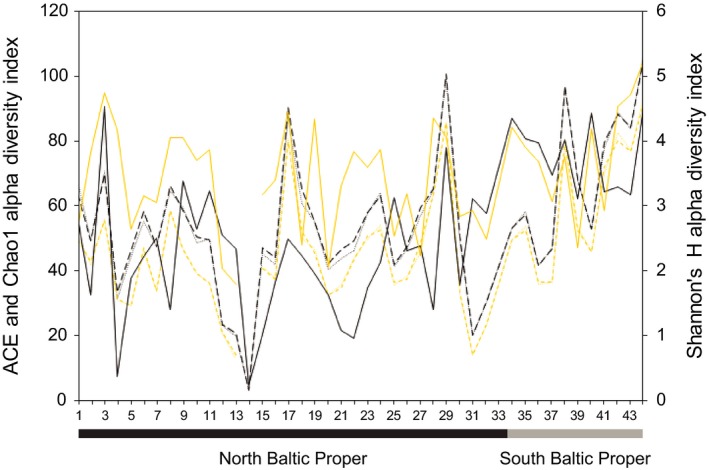
ACE, Chao1, and Shannon's *H* alpha diversity indexes of all meiofauna sequence variants (black lines) and only the Nematoda data (yellow lines). The *x*‐axis shows the station numbers (Figure 1). The line type denotes: dashed lines, ACE; dotted lines, Chao1; and filled lines, Shannon's *H* [Colour figure can be viewed at http://wileyonlinelibrary.com]

A higher alpha diversity, based on all Metazoa sequence variants, was observed in the SBP stations compared to the NBP (*p* < .01 for all indexes (ACE, Chao1, and Shannon's *H*); One‐way ANOVA; Figure 3). When alpha‐diversity was tested on the Nematoda sequence variants alone, there was also a significant difference (*p* < .01 for ACE and Chao1, *F* = 4.1 for both; Shannon's *H* not significant; Figure 3). Similar results for the nematodes were also observed when ACE was tested on non‐subsampled data (*p* < .01), although not when all metazoa sequence variants were tested (*p* = .08). These results show that a higher diversity of Metazoa sequence variants were obtained in SBP sediments. A full list of alpha‐diversity indexes for each station for all meiofauna and Nematoda sequence variants is available in Table S4.

### 3.2 Meiofauna community composition

**Figure 4:**
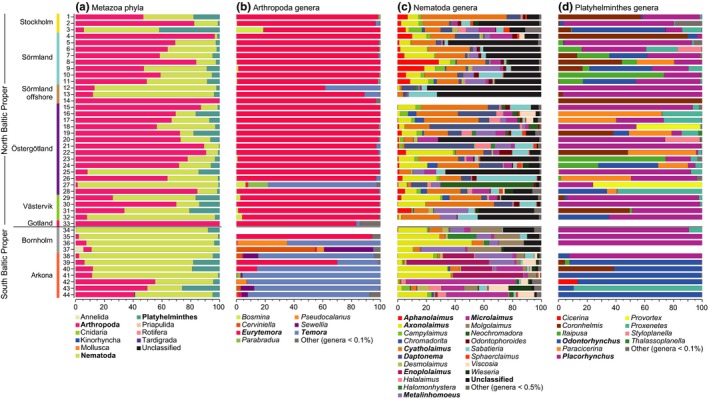
The figure shows stacked bars of the 18S rRNA gene meiofauna dataset in the north and south Baltic Proper 0–2 cm sediment layer, as well as their specific geographic regions. The *y*‐axis shows the station number, and (a) shows relative abundance (%; *x*‐axis) of Metazoa phyla; (b) genera in the Arthropoda; (c) Nematoda; and (d) Platyhelminthes phyla. Bolded text denotes major phyla or genera for each respective graph [Colour figure can be viewed at http://wileyonlinelibrary.com]

Similar to the NMDS and alpha diversity analysis, there was a difference in relative abundance in phyla between the NBP and SBP, with Arthropoda having a higher relative abundance in the NBP compared to the SBP (*p* < .01, Mann–Whitney *U* test). In contrast, the phylum Nematoda had a lower relative abundance in the NBP (*p* < .01, Mann–Whitney *U* test; Figure 4a). Looking closer at the genera belonging to Arthropoda, the genus *Eurytemora* was dominant in the NBP compared to the SBP where *Temora* had the highest relative abundance (*p* < .01 for both, Mann–Whitney *U* tests; Figure 4b).

Nematodes showed a much higher diversity compared to the other major phyla, with 60 Nematoda genera compared to 28 and 19 genera belonging to Arthropoda and Platyhelminthes, respectively (Figure 4c, a full list of all genera is available in Table S2). The phylogenetic placement of Nematoda sequences on a reference tree showed that the most dominant Nematoda sequences (Table S1) aligned closely to NCBI reference sequences from Holovachov et al. (2017) (Figure S3). The Nematoda results also indicates that NMDS ordination of Bray‐Curtis dissimilarities and homogeneity of variance between geographic regions show near‐identical results as the meiofauna data set without pelagic Arthropoda (Figures S2a,b) (Nematoda results are available in Figure S4 and Table S3), suggesting that Nematoda were key organisms affecting meiofaunal community composition. Looking closer at the Nematoda genera there was a significant higher relative abundance for *Aphanolaimus*, *Cyatholaimus*, and *Daptonema* in the NBP compared to the SBP (all *p* < .01, Kruskal–Wallis test; Figure 4c). In contrast, the genera *Axonolaimus* and *Enoplolaimus* had a higher relative abundance in the SBP (*p* < .05 and *p* < .01, respectively; Kruskal–Wallis test; Figure 4c). In addition, the relative abundance of unclassified sequence variants belonging to the Nematoda phylum was higher in the NBP (*p* < .01, Kruskal–Wallis test). The relative abundance of Nematoda unclassified sequence variants was especially high in the Sörmland regions (Figure 4c). The phylogenetic placement analysis indicated that the most relatively high abundant unclassified Nematoda sequences belonged to the genus *Chromadorita* (Table S1 and Figure S3).

**Figure 5:**
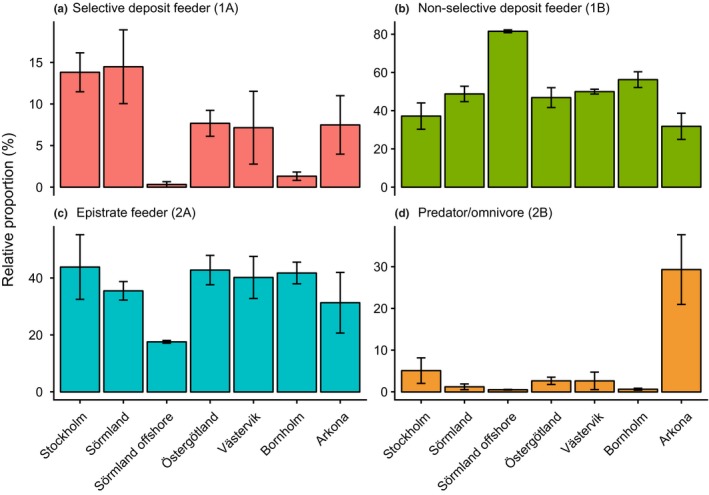
The figure shows the four Wieser (1953) nematode feeding types of the Nematoda genera for each region (classification ID in parentheses). Because unclassified data could not be included in the analysis the relative proportion were based on annotated genera. Each region consist of replicates (i.e., stations) according to the Nematoda data shown in Figure 4. Note the different scale on the *y*‐axes. The error bars shows the *SE* [Colour figure can be viewed at http://wileyonlinelibrary.com]

Maturity index calculations, used to estimate nematode genera as habitat colonizers or persisters, showed that all observed nematode genera in the current study are classified closer to colonizers rather than persisters (maturity index < 2.7; Table S5). In more detail, values closer to one indicate colonizers with high reproduction able to more easily colonize new habitats, while values closer to five indicate persisters with slow reproduction (Bongers, 1990). Nematode genera were also classified into feeding type (according to Wieser, 1953), and showed that the most southern region Arkona had more predators/omnivores compared to all other regions (one‐way ANOVA Tukey HSD post hoc test, *p* < .01; Figure 5d). Looking at the feeding types of nematode genera with a high relative abundance in the NBP the *Cyatholaimus* and unclassified sequence variants (potentially *Chromadorita*) were classified as epistrate feeders (feeding type 2A) (Table S5; unclassified sequence variants not included). In the SBP the genera *Enoplolaimus* was classified as predatory possessing large teeth (2B), while *Microlaimus* was classified as 2A (Table S5). Other genera with a high relative abundance in the Nematoda data set such as *Aphanolaimus*, *Daptonema*, and *Axonolaimus* were classified as type 1A or 1B, being either selective or nonselective deposit feeders, respectively. A full list of maturity indexes and feeding type classifications is available in Table S5.

Looking at the Platyhelminthes the genus *Odontorhynchus* showed a significant difference with a higher relative abundance in the SBP, although with high variation (*p* < .05, Mann–Whitney *U* test; Figure 4d). In the two SBP regions the genus *Placorhynchus* was dominant in the Bornholm region while *Odontorhynchus* was more prevalent in the Arkona region (*p* < .05, Mann–Whitney *U* test).

### 3.3 Macrofauna in the sediment

**Figure 6:**
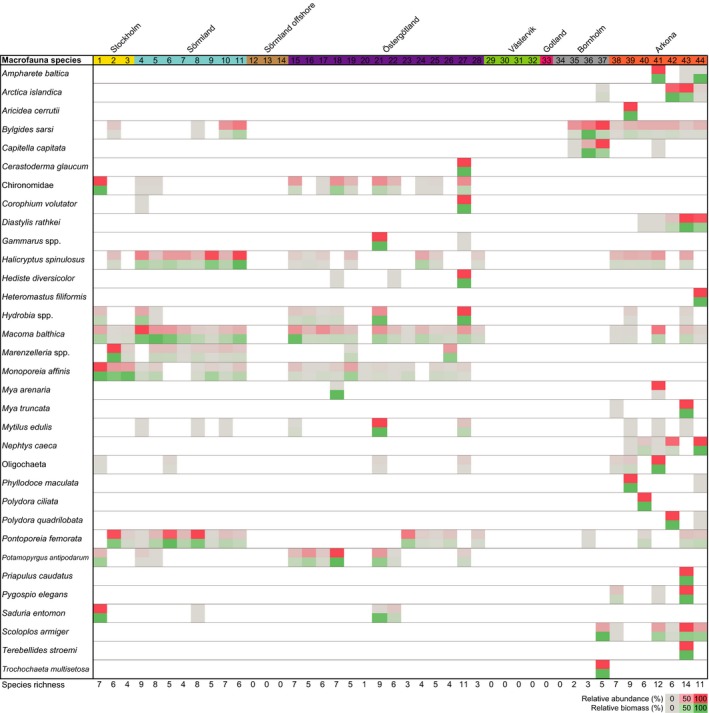
The heatmap shows collected macrofauna from the sieved sediment. The stations are numbered and region coloured on the top *x*‐axis. Species level are shown for most macrofauna, except for the class Oligochaeta and family Chrinonomidate. The grey–red gradient shows the relative proportion per species (%) of abundance per m^2^ sediment, while the grey–green gradient shows relative proportion per species of g wet weight biomass per m^2^ sediment. The species richness are shown on the bottom *x*‐axis [Colour figure can be viewed at http://wileyonlinelibrary.com]

The Macrofauna data showed a higher species richness in the SBP than in NBP (on average eight species per station compared to four in the NBP; Figure 6). There were also more species belonging to the Annelida phylum in the SBP, e.g., *Bylgides sarsi*, *Nepthys caeca*, *Pygospio elegans*, and *Scoloplos armiger* (Figure 6). The Bornholm region had the lowest macrofauna richness, with an average of three macrofauna species per station, including the Mollusca *Arctica islandica*, and two Annelida species *B. sarsi* and *Capitella capitata* (Figure 6). In contrast, other species were only present in the NBP e.g., the Amphipod *Monoporeia affinis* and Isopod *Saduria entomon* (Figure 6). Macrofauna were found at almost all stations, except in three regions (Sörmland offshore, Västervik, and Gotland; Figure 6). A full list of measured values, i.e., not relative proportions, of abundance per m^2^ sediment and gram wet weight biomass per m^2^ sediment are presented in Table S6.

### 3.4 Abiotic variables

Bottom water salinity increased as expected in the Baltic Sea (Ojaveer et al., 2010), from 5.3 ppt salinity in the NBP to 18.9 ppt in the SBP (Table 1). Bottom water temperature was generally low for most stations (average of ~6°C) except a few stations in the Östergötland region that had temperatures >10°C (average of ~11°C, stations 16–20; Table 1). Dissolved oxygen was lower in the stations located in the SBP (~6 mg/L; stations 34–44) compared to the NBP (~9 mg/L). However, only the deepest stations in the data set had oxygen concentrations that could be considered hypoxic/anoxic (stations 12, 14, 32, and 33 at 79, 124, 79, and 112 m water column depth; Table 1). Sediment OM was on average ~12.6% for all stations, but especially higher in the Östergötland regions that had ~16% (stations 15–28; Table 1).

**Table 1:** List of the station numbers, region, date of sampling during 2015, latitude, longitude, and water column depth

Station	Region	Date	Lat. (dd)	Long. (dd)	Depth (m)	Salinity (ppt)	°C	O_2_ (mg/L)	WC (%)	OM (%)
1	Stockholm	27 May	59.5243	18.8533	23.5	5.3	9.0	10.7	86.1	14.0
2	Stockholm	27 May	59.5081	19.0044	58.5	6.6	5.0	8.8	62.7	4.6
3	Stockholm	27 May	59.4788	18.9215	40.3	5.8	6.0	10.9	68.1	6.0
4	Sörmland	17 May	58.8408	17.5518	22	6.3	7.7	11.1	77.0	11.7
5	Sörmland	19 May	58.8261	17.5761	39	6.6	5.0	10.7	81.5	12.2
6	Sörmland	17 May	58.8109	17.6069	37.5	6.7	4.7	10.9	82.2	12.5
7	Sörmland	16 May	58.7902	17.7284	38	6.7	4.4	10.5	80.3	9.9
8	Sörmland	17 May	58.7740	17.6914	44	6.7	4.5	11.0	68.1	6.2
9	Sörmland	16May	58.7669	17.8313	53	6.9	4.1	11.0	73.8	7.3
10	Sörmland	16 May	58.7440	17.8140	47	6.9	4.2	10.4	79.7	9.9
11	Sörmland	16 May	58.7189	17.8423	59	7.0	4.3	10.4	68.8	6.2
12	Sörmland offshore	07 May	58.5674	17.9085	79	9.1	5.4	0.3	86.2	12.2
13	Sörmland offshore	07 May	58.5489	18.0253	78	6.5	4.9		79.8	7.7
14	Sörmland offshore	07 May	58.4941	18.1167	124	9.9	5.4	0.0	93.5	18.7
15	Östergötland	01 June	58.3961	16.8854	14	6.3	8.7	9.3	86.8	15.0
16	Östergötland	01 June	58.3791	16.9711	12.5	6.4	11.1	10.3	85.5	14.9
17	Östergötland	01 June	58.3763	16.9808	13.5	6.5	10.9	10.4	87.3	16.8
18	Östergötland	01 June	58.3739	16.9444	10	6.4	12.3	10.1	87.8	17.0
19	Östergötland	01 June	58.3697	16.9604	16	6.4	11.1	10.3	85.5	15.4
20	Östergötland	01 June	58.3621	16.9433	19.5	6.4	11.4	10.1	92.1	19.1
21	Östergötland	01 June	58.3234	16.9364	15.6	6.6	7.5	10.5	85.9	14.8
22	Östergötland	02 June	58.3220	16.9715	20.5	6.6	6.7	10.7	89.0	18.6
23	Östergötland	02 June	58.2543	16.7866	39	6.8	6.1	9.8	87.8	15.7
24	Östergötland	02 June	58.2249	16.8153	25	6.7	6.6	10.5	84.7	14.3
25	Östergötland	02 June	58.2169	16.8432	30	6.7	5.6	10.9	85.7	13.9
26	Östergötland	02 June	58.2095	16.9378	33	6.7	5.8	10.9	87.0	18.8
27	Östergötland	02 June	58.2027	16.9152	9.6	6.6	8.6	10.6	87.9	16.4
28	Östergötland	02 June	58.1980	16.8501	29.1	6.7	6.1	10.8	81.3	12.1
29	Västervik	08 May	57.7334	17.0916	72	8.5	4.8	1.5	90.0	16.9
30	Västervik	08 May	57.6019	17.0010	67	7.6	4.5	6.5	71.9	6.3
31	Västervik	08 May	57.5252	16.9691	66	7.7	4.5	6.9	90.1	18.4
32	Västervik	08 May	57.4763	17.0633	79	8.8	5.1	0.0	76.4	7.3
33	Gotland	14 May	57.4000	19.3498	112	11.0	6.2	0.1	94.3	24.7
34	Bornholm	09 May	55.7502	15.9332	64	16.0	7.6	4.7	84.6	12.7
35	Bornholm	12 May	55.6668	16.0658	71	17.2	7.5	2.7	82.0	10.8
36	Bornholm	09 May	55.6177	14.8630	80	18.3	7.2	3.7	85.4	12.7
37	Bornholm	12 May	55.2507	15.9888	91	18.9	7.1	2.8	86.2	13.6
38	Arkona	10 May	55.2334	13.3334	41	14.0	5.5	6.9	69.2	6.6
39	Arkona	10 May	55.2246	13.4182	42	13.9	5.5	7.2	69.5	10.0
40	Arkona	10 May	55.2250	13.6335	43	13.4	5.6	8.6	83.7	13.0
41	Arkona	10 May	55.2248	13.2667	40	14.0	5.6	6.2	76.3	10.1
42	Arkona	10 May	55.1333	13.6666	45	14.3	5.6	8.3	84.2	13.6
43	Arkona	10 May	55.1239	13.2615	40	12.4	5.9	8.8	56.0	4.5
44	Arkona	12 May	55.0090	14.0738	48	14.9	5.5	7.2	83.9	13.3

Note

Abiotic parameters measured include bottom water salinity, temperature, dissolved oxygen (mean of two technical measurements), percentage of sediment water content (WC), and sediment organic matter (OM) content. Missing data is denoted by an empty cell.

### 3.5 Correlations of meiofauna with abiotic variables and macrofauna data

**Figure 7:**
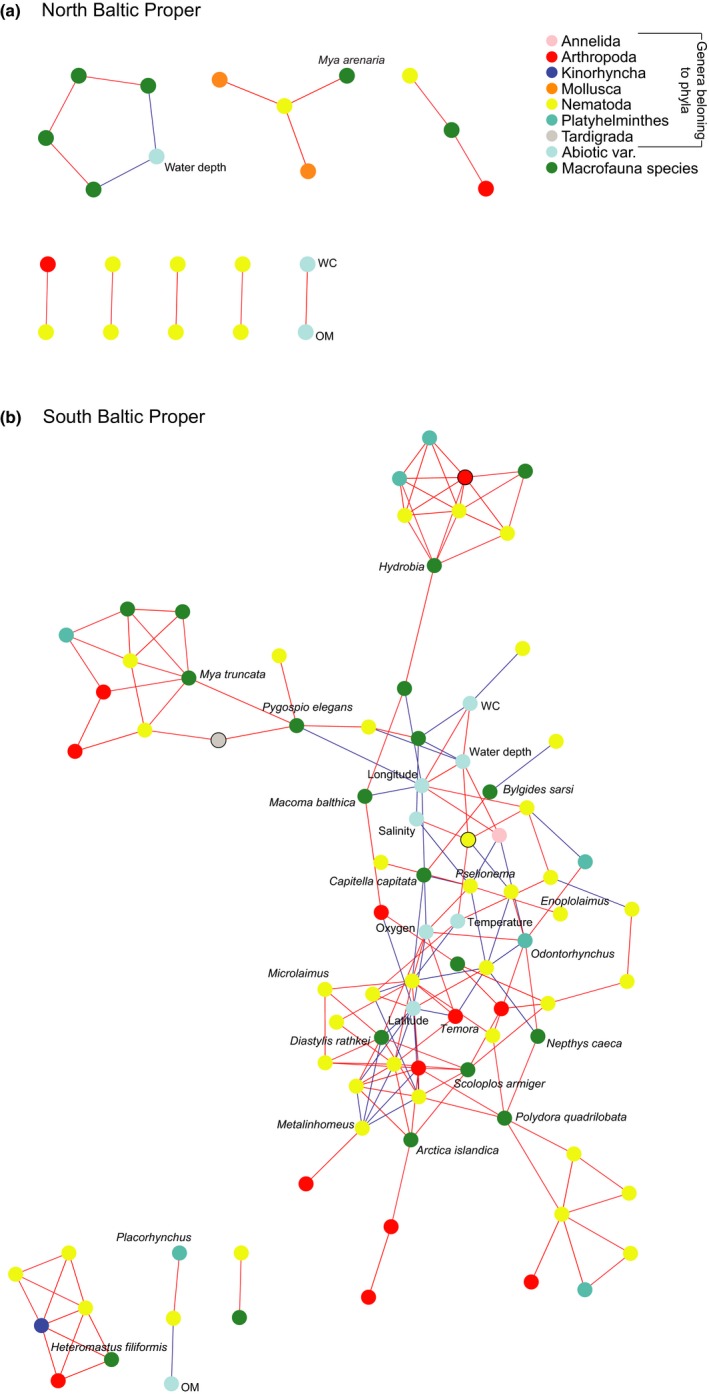
Correlation networks of spearman correlations based on data from north (a) and south Baltic Proper (b). The correlations included meiofauna 18S rRNA gene data (each node represents one Metazoa genus), abiotic variables, and macrofauna abundance data. The mean was used for the two oxygen technical replicates. The colour of the lines denote *ρ* ≥ 0.7 (red) or ≤–0.7 (blue). All correlations are statically significant (*p* < .05). All abiotic nodes have been labelled as well as a few genera/macrofauna nodes according to the results presented in the text. Nodes with black borders denote unclassified sequences belonging to a certain phylum [Colour figure can be viewed at http://wileyonlinelibrary.com]

Abiotic data from all stations were tested with Bray‐Curtis dissimilarity of sequence variants, and the best explainable abiotic variables were longitude, latitude and salinity (*ρ* = 0.73). Mantel tests also confirmed that these abiotic variables were significantly correlated with the beta diversity measures (*R* ^2^ = 0.67 and *p* < .01, for both salinity and spatial location tested). The combination of abiotic variables latitude, sediment water content, and oxygen had the best rank correlation explaining the beta diversity among the stations in the SBP (*ρ* = 0.57; “bioenv” test in r package vegan). This was in contrast to the NBP where longitude, water depth, and oxygen were the best explainable variables (although with a low rank correlation, *ρ* = 0.32; in accordance to the lack of correlations with abiotic factors in the correlation network; Figure 7a).

Correlation network analysis was conducted on the NBP and SBP separately because the NDMS Bray‐Curtis indicated differences in meiofaunal community structure between the two areas. In addition, the bioenv analysis showed salinity to be a strong driver influencing meiofaunal community structure and diversity in the Baltic Proper. This precaution of removing sample heterogeneity in a larger ecosystem‐wide data set is in accordance to Röttjers and Faust (2018) to lower the risk of unwanted effects on correlation network analysis. Because the macrofauna abundance per m^2^ values correlated strongly with their biomass per m^2^ data (*ρ* = 0.74, *p* < .01; all abundance and biomass values tested together, *n* = 220), for conciseness only the abundance m^‐2^ data were used in the correlation network analysis.

The NBP did not show any major significant correlations with the dominant Metazoa genera observed in Figure 4, i.e., Arthropoda, Nematoda, and Platyhelminthes (Figure 7a). The Nematoda phyla *Axonolaimus* were correlated with Nematoda *Odontophoroides*, and two Mollusca and the macrofauna species *Mya arenaria* formed a cluster of correlations with low abundant nematodes and arthropods (Figure 7a), while a few other macrofauna species correlated negatively with water depth (e.g., Chironomidae, *Macoma balthica*, and *Hydrobia*; Figure 7a; correlation networks with all labels shown are available in Figures S5 and S6 for NPB and SBP, respectively). In contrast, the SBP showed a complex web of significant correlations between chemistry, macrofauna and especially Nematoda genera (Figure 7b). This difference between the NBP and SBP was also confirmed when all Metazoa sequence variants were tested for correlations (i.e., not tested on taxonomical genera level; Figures S7 and S8, respectively). In the SBP abundant Nematoda genera *Microlaimus* correlated positively with several other nematode genera and the macrofauna crustacean species *Diastylis rathkei* (Figure 7b). The predator *Enoplolaimus*, a nematode with one of the highest relative abundance in the SBP, correlated positively with the low abundant Nematoda genera *Pselionema* (Figure 7b). The predator *B. sarsi* that was one of the few macrofauna species in the Bornholm region correlated negatively with the Nematoda genus *Campylaimus* (Figure S7). Other correlations included e.g., Nematoda genera with other Nematoda, and the Arthropoda genus *Temora* with macrofauna and Nematoda (Figure 7b). In addition, Crustacean genera were correlated with Nematoda and oxygen (Figure 7b), and the Platyhelminthes genera *Odontorhynchus* was associated with several Nematoda genera and the macrofauna *N. caeca*. Mollusca species such as *A. islandica*, *Mya truncata*, and *Hydrobia* were found in a few clusters involving various meiofauna genera. Finally, a few Annelida macrofauna species such as *P. elegans*, *Polydora quadrilobata, and Heteromastus filiformis* formed the beginning, or were part of correlation clusters associated with low abundant meiofauna genera (Figure 7b).

## 4 DISCUSSION

### 4.1 Abiotic explanatory variables of meiofaunal diversity

Salinity was the major explanatory variable of benthic meiofauna community composition in the Baltic Proper. In addition to our findings, salinity has been observed to influence macrofauna in the Baltic Sea (Gogina et al., 2016) and meiofauna community structure studied elsewhere (Coull, 1988; Lallias et al., 2014). Interestingly, similar findings were also recently discovered for sediment bacteria community composition along a salinity transect in the Baltic Sea (Klier, Dellwig, Leipe, Jürgens, & Herlemann, 2018). Bottom water oxygen also correlated with the difference in meiofaunal community composition, especially in the SBP. The role of oxygen is not surprising considering that oxygen is essential for the majority of meiofaunal organisms (Braeckman et al., 2013), and oxygen availability is known to cause shifts in the community composition of for example nematodes (Nguyen et al., 2018). The local regions as defined in this study (Figure 1 and Table 1), also harboured significantly different communities of meiofauna (Figures 2 and 4). This difference could be attributable to specific salinity preferences, but also due to the sediment substrate and available food resources (Lee, Tietjen, Mastropaolo, & Rubin, 1977), and adult dispersal through water currents (Hagerman & Rieger, 1981). Marine meiofaunal communities have previously been shown to be heterogeneous both at large (Fonseca et al., 2014) and small spatial scales (Findlay, 1981). Our results indicate salinity to be a major barrier to dispersion of meiofauna species in the Baltic soft sediment, by limiting the dispersion of marine species to the north and of freshwater species to the south. Limitation to dispersion is an important factor driving community assembly in ecological systems (Vellend, 2010). Therefore the salinity gradient in the Baltic Sea influences sediment habitats with different kinds of food and predators that will in turn influence the meiofauna community composition and diversity.

### 4.2 Geographical differences in community composition

Meiofaunal diversity was dominated by a large variety of Nematoda genera. This was not surprising considering that nematodes are highly diverse (Zhang, 2013), and typically the most abundant meiofauna found in the sediment surface (Coull, 1999). The SBP had a different Nematoda community composition, probably due to the higher salinity conditions that have previously been found to influence diversity and community structure in the Baltic Sea (Ojaveer et al., 2010). In the NBP there was a large proportion of unclassified Nematoda sequences (Figure 4c) and could possibly be due to the lack of freshwater‐brackish species being classified in the reference databases (Holovachov et al., 2017). These unclassified sequences were indicated in the phylogenetic placement analysis to be affiliated with the genus *Chromadorita* (Figure S3). This genus has previously been found in the Baltic Sea (Jensen, 1979) and contains species living on macrophytes (Jensen, 1979), free‐living and feeding on diatoms (Jensen, 1984), and living inside cyanobacterial biofilms (Gaudes, Sabater, Vilalta, & Muñoz, 2006). The most southern region Arkona had not only a higher diversity but also a higher proportion of nematode predators/omnivores (Figure 5d), which could explain why there were more ecological correlations in the SBP (Figure 7b). Even though the PCR primers used might have selected for certain eukaryotic species, and primer bias are likely to pervade all metabarcoding studies, we used the same biodiversity discovery method (i.e., metabarcoding primers) throughout. Despite the imperfect nature of metabarcoding (and other ecological sampling approaches), the difference among regions and areas in the Baltic Proper were statistically significant and showed stark dissimilarities in community composition.

In addition to nematodes, there was also a large relative abundance of Arthropods in the 18S rRNA gene data set, especially in the NBP. The majority of the Arthropoda belonged to the pelagic copepod genera *Eurytemora* and *Temora* in the NBP and SBP, respectively (Figure 4a,b). The hatching rate and development time of e.g., *Eurytemora affinis* is negatively affected by low salinity (Karlsson, Puiac, & Winder, 2018) which can explain the difference between the north and south regions. Possible additional explanations for copepods being in the sediment could be due to sinking marine snow containing carcasses, resting stages such as buried eggs or dormancy (Dahms, 1995). The high relative abundance of Arthropoda could therefore be derived from DNA being extracted from large amounts of copepod eggs or resting stages buried in the sediment surface. Considering that similar results have also been observed by Nascimento et al. (2018) from sediments collected in the Stockholm region, the large proportion of copepods is probably a trait for low saline waters (<10 ppt) in the Baltic Sea. Compared to the SBP where salinity is higher, the availability of copepod eggs in the low‐saline NBP can be a larger source of energy for benthic macrofauna populations (Karlson & Viitasalo‐Frösen, 2009). In addition, because the hatching rate is slower in low salinity (Karlsson et al., 2018) the accumulation of a seed bank followed by subsequent hatching could enhance the benthic‐pelagic coupling. Our results highlight important geographic differences in meiofaunal communities that are only possible to uncover with modern molecular tools (Fonseca et al., 2010).

### 4.3 Biotic interactions

Macrofauna species richness and meiofauna diversity were both higher in the SBP (Figures 6 and 3, respectively). Nascimento et al. (2011) found that a higher species richness of macrofauna increased interference competition among meiofauna and/or limited food availability in a laboratory study. Potentially, this could partly explain why there were more ecological connections between macro‐ and meiofauna in the SBP as indicated by the correlations network data (Figure 7b). On the other hand, macrofaunal bioturbation can create more habitable niches and higher variety of food types allowing for a higher meiofauna diversity (Meysman, Middelburg, & Heip, 2006). The significant correlations included mainly Annelida as well as crustacean macrofauna, which are well‐known bioturbators (Krantzberg, 1985). In addition, bottom water oxygen was one of the central nodes in the correlation network with connections to meio‐ and macrofauna (Figure 7b). It is therefore possible that oxygen rich burrows made by annelids (Aller, 1988) or other modes of bioturbation by macrofaunal organisms (Krantzberg, 1985) stimulate bacterial growth and make specific niches and habitats favourable for meiofauna (reviewed in Olafsson, 2003). However, negative macro‐meiofauna interactions have also been previously reported (reviewed in Olafsson, 2003). High macrofauna diversity can increase sediment oxygen consumption (Bolam, Fernandes, & Huxham, 2002), and interference competition with meiofauna by limiting its access to freshly deposited detritus (Nascimento et al., 2011). Such mechanism could explain some of the negative or nonsignificant correlations between macro‐ and meiofauna taxa found in our study. For example, we observed several Mollusca macrofauna in correlation clusters with meiofauna genera in the SBP (Figure 7b). However, this kind of interaction was not as prominent in the NBP. This is in accordance with previous experimental studies with sediments from the Sörmland region amended with bivalve *M. balthica* that showed no significant difference on the majority of meiofauna, including nematodes (Olafsson, Elmgren, & Papakosta, 1993). Considering that correlation network analysis can be a major strength to visualize and detect specific habitat niches (Röttjers & Faust, 2018), the meiofauna‐macrofauna associations observed here could be indirect effects of shared niche preference. In addition, predation is an important mechanism structuring diversity in more stable and tropically complex communities (Menge & Sutherland, 1976). The NBP had lower diversity and has a history of being more affected by eutrophication compared to the southern region Arkona (Andersen et al., 2015). The higher relative abundance of nematode predators in the SBP (Figure 5d) could indicate a relatively more stable environment where predation can maintain a higher diversity helped by more macrofauna‐mediated niches, biodiversity and interactions. Although, network correlations based on metabarcoding data need to be treated with caution (see Röttjers & Faust, 2018), our results clearly indicate that there are fewer, direct or indirect associations between meiofauna and macrofauna in low‐saline areas in the Baltic Sea.

### 4.4 Effects of climate change and future scenarios

The area of low saline regions in the Baltic Sea (surface water salinity < 6 ppt) has increased since the 1970s and are predicted to further increase with climate change due to elevated levels of runoff (Vuorinen et al., 2015). As indicated here a decrease in salinity might be accompanied by a decrease in meiofaunal biodiversity and biotic interactions in the Baltic Sea. Salinity strongly influences the community composition and diversity in other coastal systems (Lallias et al., 2014; Van Diggelen & Montagna, 2016) where similar effects can happen if salinity is reduced as a consequence of climate change. Additionally, it is clear from our results that a continued expansion of hypoxic bottom zones will significantly alter benthic community structure. This may influence important ecosystem functions regulated by meiofauna, like OM degradation and nutrient cycling. Here, we show that multiple anthropogenic pressures like eutrophication (Finni, Kononen, Olsonen, & Wallström, 2001), expansion of hypoxic bottom zones (Meier et al., 2011), and of low‐salinity areas (Vuorinen et al., 2015), will probably have profound impacts on benthic communities of anthropogenically stressed coastal systems. Ongoing environmental change will lead to lower benthic biodiversity and fewer biotic interactions. Such structural changes to benthic community composition will probably influence ecosystem functions and services, and decrease ecosystem stability (McCann, 2000).

## AUTHOR CONTRIBUTIONS

E.B. analysed data and drafted the manuscript, C.R. sampled in the field and together with C.S. conducted laboratory work. J.G. helped with field sampling and gave feedback on the manuscript, S.C. helped designed the study and gave feedback on the manuscript. F.N. designed the study, conducted laboratory work, analysed data and contributed to the manuscript writing. All authors gave final approval for publication.

## Data Availability

The raw sequence data have been uploaded and are available on the NCBI database with the following BioProject number: PRJNA497177.
