# Supplementary material for: Salinity drives meiofaunal community structure dynamics across the Baltic ecosystem
Source: Mol Ecol. 2019 Sep 5;28(16):3813–29. doi: 10.1111/mec.15179 (PMC6852176; doi:10.1111/mec.15179)
Supplement: Supplementary file 1 [file MEC-28-3813-s001.pdf]

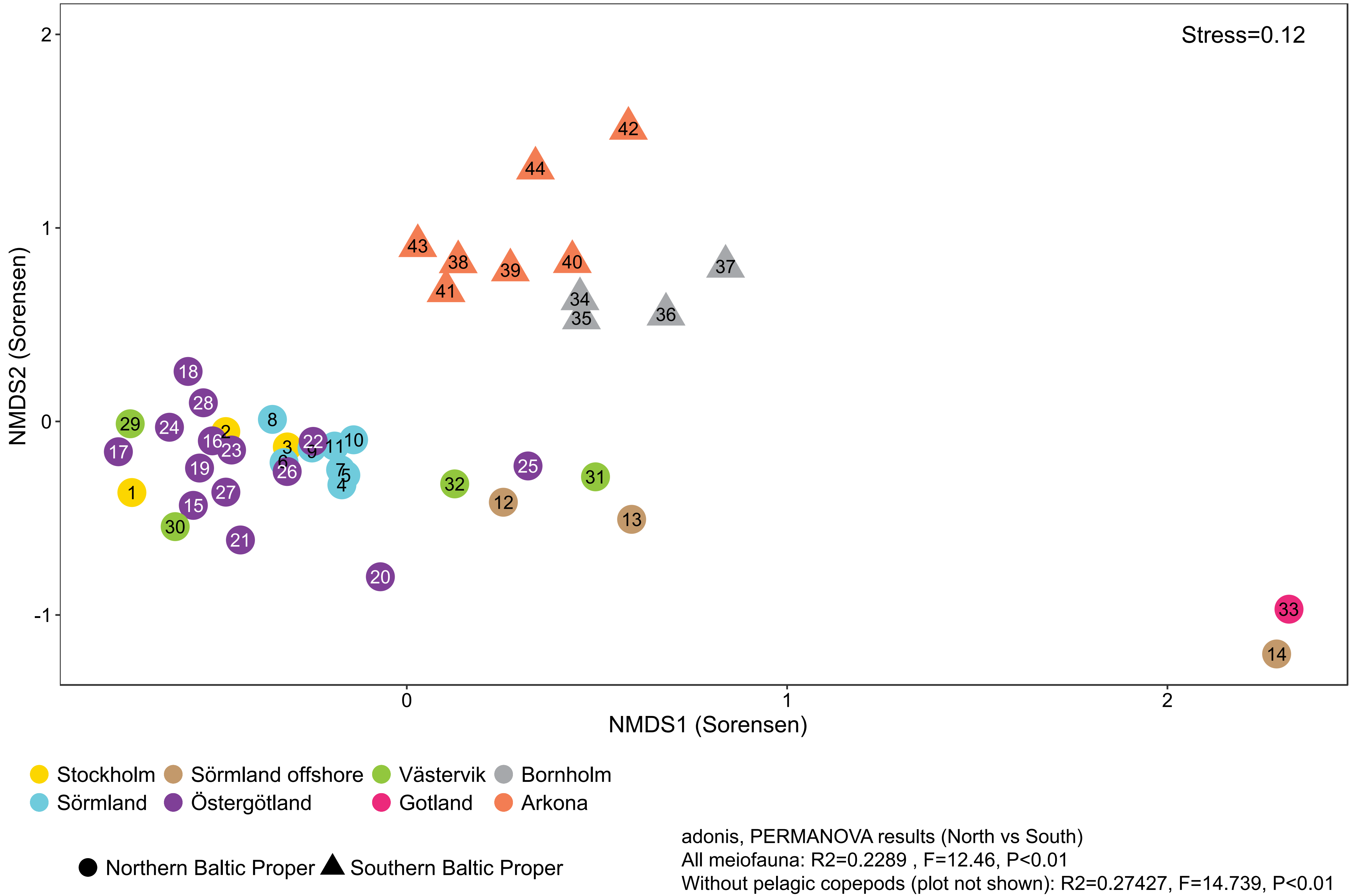
Stress=0.12
NMDS1 (Sorensen)
NMDS2 (Sorensen)
Stockholm
Sörmland offshore
Västervik
Bornholm
Sörmland
Östergötland
Gotland
Arkona
Northern Baltic Proper
Southern Baltic Proper
adonis, PERMANOVA results (North vs South)
All meiofauna: R2=0.2289 , F=12.46, P<0.01
Without pelagic copepods (plot not shown): R2=0.27427, F=14.739, P<0.01
