# Supplementary figures and images for: Salinity drives meiofaunal community structure dynamics across the Baltic ecosystem
Source: Mol Ecol. 2019 Sep 5;28(16):3813–29. doi: 10.1111/mec.15179 (PMC6852176; doi:10.1111/mec.15179)

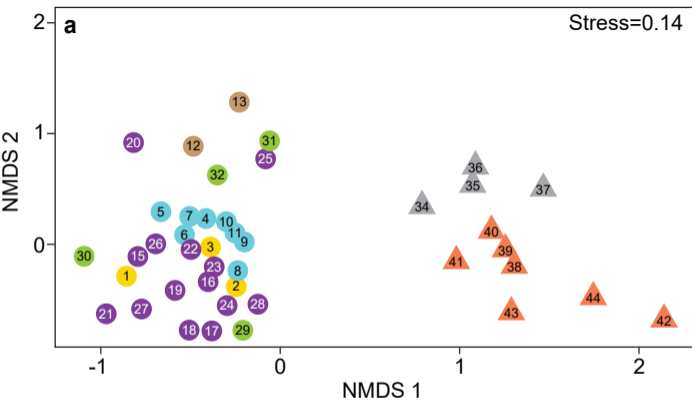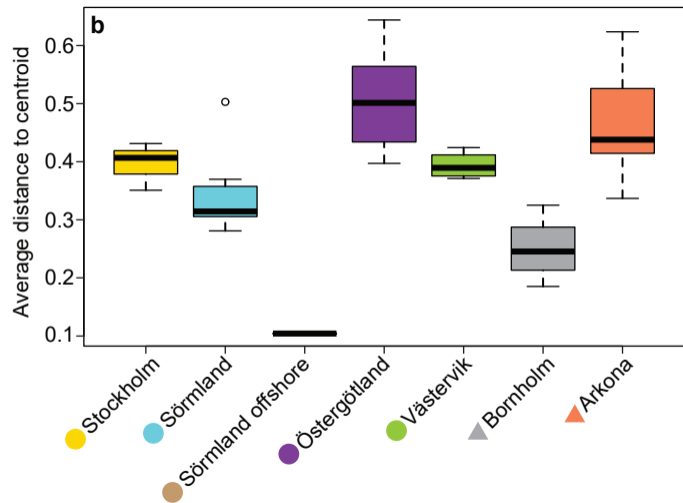

Supplement: Supplementary file 2 [file MEC-28-3813-s002.pdf]

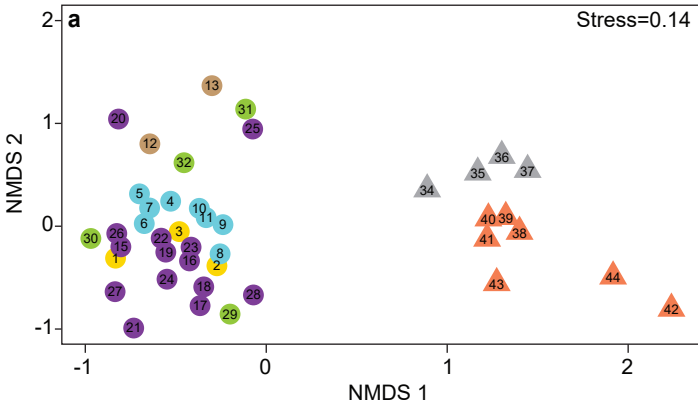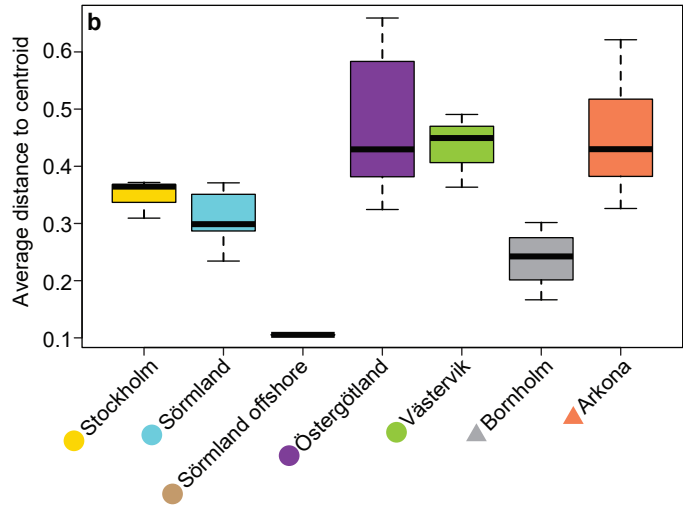

Supplement: Supplementary file 4 [file MEC-28-3813-s004.pdf]

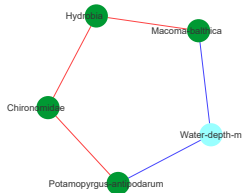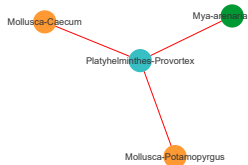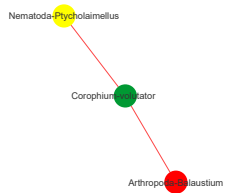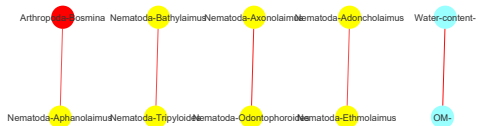

Supplement: Supplementary file 5 [file MEC-28-3813-s005.pdf]

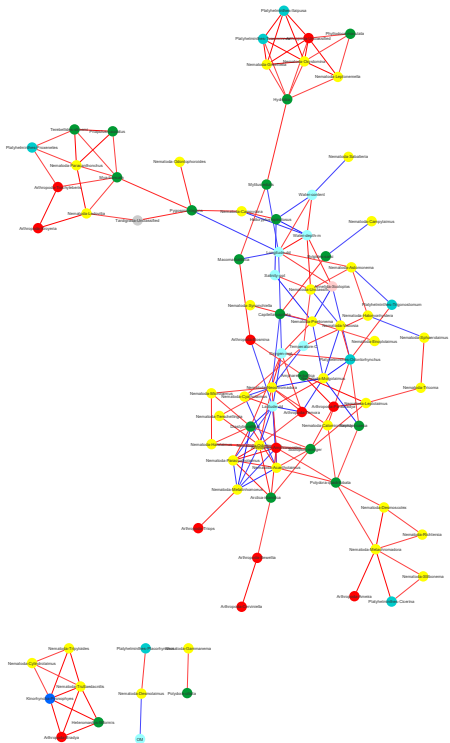

Supplement: Supplementary file 6 [file MEC-28-3813-s006.pdf]

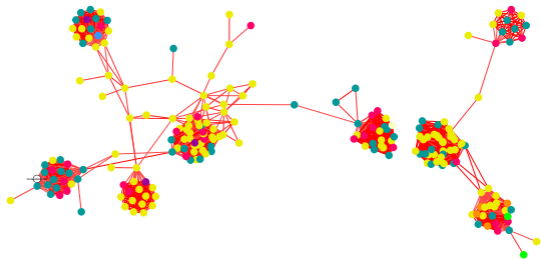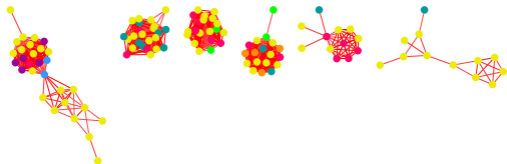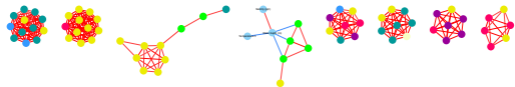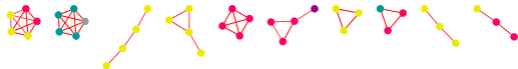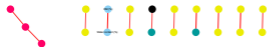

Supplement: Supplementary file 7 [file MEC-28-3813-s007.pdf]

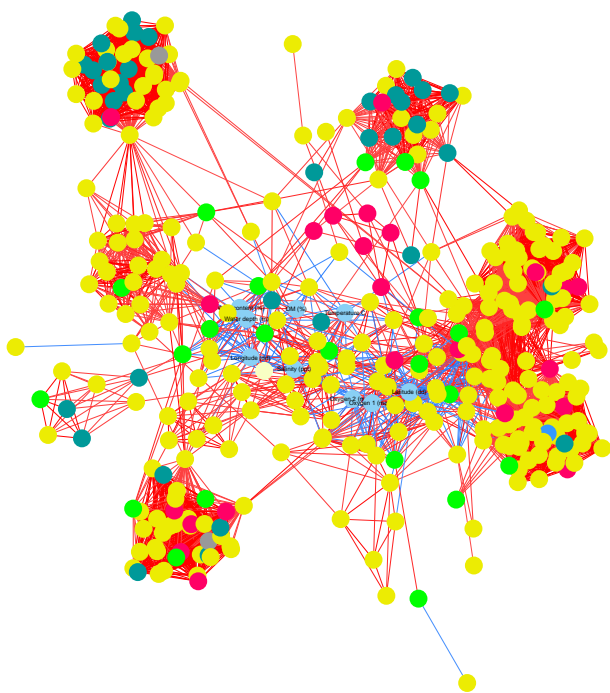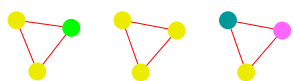

Supplement: Supplementary file 8 [file MEC-28-3813-s008.pdf]
