# Supplementary material for: Salinity drives meiofaunal community structure dynamics across the Baltic ecosystem
Source: Mol Ecol. 2019 Sep 5;28(16):3813–29. doi: 10.1111/mec.15179 (PMC6852176; doi:10.1111/mec.15179)
Supplement: Supplementary file 15 [file MEC-28-3813-s015.docx]

**Text S1. PCR protocol**

For the 18S rRNA gene marker, use primers 18S_TAReuk454FWD1f and 18S_TAReukREV3r.

**Preparing PCR 1 - Mastermix**

Q5 HS High-Fidelity Master Mix (2X)^*^ 12.5µl

Round 1 Primer F (10 μM) 0.5µl

Round 1 Primer R (10 μM) 0.5µl

PCR Water 10.5µl

Final Vol per reaction: 24µl

* New England Biolabs (USA)

For each sample mix 24 μl of the MasterMix and 1 μl of DNA template.

**PCR 1 - program**

98º C 30s

15 cycles (98º C 10s; 50º C 30s; 72º C 30s)

72º C 10 min

4º C

**Cleanup PCR 1 products – preparing cleanup Mastermix**

Exonuclease I 0.1 µl

TSAP 0.2 µl

Mol Biol Water 0.7 µl

Final Vol per sample: 1 µl

Mix 12 μl PCR 1 product with1 μl of the cleanup Mastermix

Put the samples in a Thermal Cycler, using the following programme:

37º C 15 min

74º C 15 min

4º C forever

This allows the enzyme activities followed by heat inactivation.

**Preparing PCR 2 - Mastermix**

Q5 HS High-Fidelity Master Mix (2X) 12.5 µl

Nextera XT Index Primer 1 (N7xx) 0.5 µl

Nextera XT Index Primer 1 (S5xx) 0.5 µl

PCR Water 6.5 µl

Final Vol per reaction: 20µl

Mix 20 μl of the MasterMix with 5 μl of purified (Exo-TSAP treated) PCR 1 product.

**PCR 2 - program**

95°C for 3 minutes

15 cycles (95°C for 30 seconds, 55°C for 30 seconds, 72°C for 30 seconds)

72°C for 5 minutes

Hold at 4°C

This is followed by purification using the Agencourt AMPure XP PCR Purification kit (Beckman Coulter).
