# Supplementary material for: Salinity drives meiofaunal community structure dynamics across the Baltic ecosystem
Source: Mol Ecol. 2019 Sep 5;28(16):3813–29. doi: 10.1111/mec.15179 (PMC6852176; doi:10.1111/mec.15179)
Supplement: Supplementary file 16 [file MEC-28-3813-s016.docx]

**Salinity drives meiofaunal community structure dynamics across the Baltic ecosystem**

Elias Broman, Caroline Raymond, Christian Sommer, Jonas S. Gunnarsson, Simon Creer, Francisco J.A. Nascimento

**Supplemental Information captions**

**Text S1.** The text contains a detailed protocol of the PCR methods used in the study (docx).

**Table S1.** The Excel file shows a table of the sequence variants obtained from the DADA2 analysis of the raw sequence data. In addition, the table also shows sequence counts, and taxonomic classifications from the SILVA 132 plus NCBI NT databases, and the BLAST percentages of identity for each hit against the NCBI NT database (xlsx).

**Table S2.** The table shows the sequence counts for each Metazoa genera, sequence counts were grouped into Metazoa genera based on taxonomic results from the NCBI NT database (.xlsx).

**Table S3.** The table shows PERMANOVA results for the multivariate homogeneity of beta diversity variance (betadisper) average distance to the centroid analysis of each geographic cluster (as shown in Fig. 2b & 2d; and Fig. S2b) (xlsx).

**Table S4.** The Excel file contains two spreadsheets with the first showing alpha diversity indexes ACE, Chao1, and Shannon’s H based on the counts from all Metazoa sequence variations (counts were sub-sampled to 2200 counts for each stations and bootstrapped 100 times, except station 33 (291 counts) that was excluded). The second spreadsheet shows similar data but only for the Nematoda sequence variants data (counts were sub-sampled to 1418 and bootstrapped 100 times). Stations that did not include any Nematoda sequences were excluded (xlsx).

**Table S5.** The table to the left shows a list of annotated Nematoda genera and their maturity index and feeding type classification according to Bongers (1990) and Wieser (1953), respectively. The table to the right shows the calculated maturity index based on Nematoda genera relative abundance and maturity index classification (xlsx).

**Table S6**. The Excel file shows a table of collected macrofauna taxa from each sampling station (top row numbers). The abundance m^-2^ sediment values are shown in the first row for each species (red relative % gradient) while g wet weight biomass m^-2^ sediment values are shown in the second row for each species (green relative % gradient). Species level are shown for most macrofauna, except for the class Oligochaeta and family Chrinonomidate. The values in each cell shows the actual (i.e. non proportional) measured abundance and biomass (xlsx).

**Figure S1.** Multivariate NMDS based on the presence/absence Sorensen of all meiofauna. The colours of the circles denote specific regions, while the numbers denote each specific station. PERMANOVA results are shown in the lower right of the figure, and was also tested for the same dataset without pelagic copepods. Stations belonging to the NBP are denoted as circles while stations in the south are denotes as triangles (pdf)

**Figure S2**. Multivariate NMDS based on the relative abundance Bray-Curtis dissimilarities after removing pelagic arthropods (i.e. arthropods such as swimming pelagic amphipods that are usually encountered in the water column and generally described as zooplankton) from the data (a), and boxplots showing the homogeneity of beta diversity variance for each region (b). The colours of the symbols in the NMDS plots denote the specific regions, while the numbers denote each specific station. Stations belonging to the north Baltic Proper are presented as circles while stations in the south as triangles.

**Figure S3.** The tree shows Nematoda reference sequences as described in Holovachov, Haenel, Bourlat, and Jondelius (2017) (red text), and PaPaRa phylogenetic aligned Nematoda sequences from the 18S rRNA gene dataset (black text). Unclassified Nematoda sequences are coloured green in tree (pdf).

**Figure S4.** Multivariate NMDS based on the relative abundance Bray-Curtis dissimilarities was constructed based on all sequence variants classified as Nematoda (a). The colours of the circles denote specific regions, while the numbers denote each specific station. The figure to the right show boxplots of the homogeneity of beta diversity variance for each region (b). Stations belonging to the NBP are denoted as circles while stations in the south are denotes as triangles (pdf).

**Figure S5.** High resolution file showing results from the CoNet correlation network analysis based on the NBP data. The labels denote Metazoa genera (each node represents one genus), abiotic variables, and macrofauna abundance m^-2^ sediment. The colour of the lines denotes rho ≥ 0.7 (red) or ≤ -0.7 (blue). All correlations are statically significant (*P* < 0.05). Colours of the circles denote Metazoa phyla, or data type used (same colour code as in figure 7) (pdf).

**Figure S6.** High resolution file showing results from the CoNet correlation network analysis based on the SBP data. The labels denote Metazoa genera (each node represents one genus; relative abundance data), abiotic variables, and macrofauna abundance m^-2^ sediment. The colour of the lines denotes rho ≥ 0.7 (red) or ≤ -0.7 (blue). All correlations are statically significant (*P* < 0.05). Colours of the circles denote Metazoa phyla, or data type used (same colour code as figure 7) (pdf).

**Figure S7.** High resolution file showing spearman correlations (shown as a correlation network) of all Metazoa sequence variants from the NBP (each node represents one sequence variant; relative abundance data), abiotic variables, and macrofauna abundance m^-2^ sediment. The colour of the lines denote rho ≥ 0.7 (red) or ≤ -0.7 (blue). All correlations are statically significant (*P* < 0.05). Colours of the circles denote Metazoa phyla, or data type used (same colour code as figure 7), with the addition of the Priapulida (purple), Rotifera (black) and unclassified (white with black border) (pdf).

**Figure S8.** High resolution file showing spearman correlations (shown as a correlation network) of all Metazoa sequence variants from the SBP (each node represents one sequence variant; relative abundance data), abiotic variables, and macrofauna abundance m^-2^ sediment. The colour of the lines denote rho ≥ 0.7 (red) or ≤ -0.7 (blue). All correlations are statically significant (*P* < 0.05). Colours of the circles denote Metazoa phyla, or data type used (same colour code as figure 7), with the addition of the Cnidaria (pink) (pdf).

**References**

Bongers, T. (1990). The maturity index: an ecological measure of environmental disturbance based on nematode species composition. *Oecologia, 83*(1), 14-19. doi:10.1007/bf00324627

Holovachov, O., Haenel, Q., Bourlat, S. J., & Jondelius, U. (2017). Taxonomy assignment approach determines the efficiency of identification of OTUs in marine nematodes. *Royal Society Open Science, 4*(8). doi:10.1098/rsos.170315

Wieser, W. (1953). Die Beziehung zwischen Mundhohlengestalt, Ernahrungsweise und Vorkommen bei freilebenden marinen Nematoden. *Arkiv for zoologi, 4*, 439–484.
