# Supplementary material for: Salinity drives meiofaunal community structure dynamics across the Baltic ecosystem
Source: Mol Ecol. 2019 Sep 5;28(16):3813–29. doi: 10.1111/mec.15179 (PMC6852176; doi:10.1111/mec.15179)

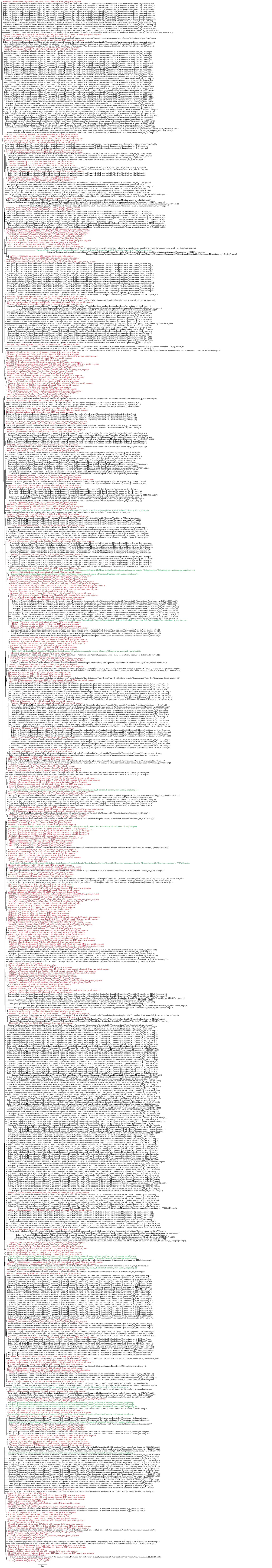

Supplement: Supplementary file 3 [file MEC-28-3813-s003.pdf]
